# Comparison of two different orthokeratology lenses and defocus incorporated soft contact (DISC) lens in controlling myopia progression

**DOI:** 10.1186/s40662-023-00358-x

**Published:** 2023-10-07

**Authors:** Na Li, Weiping Lin, Ruixue Liang, Ziwen Sun, Bei Du, Ruihua Wei

**Affiliations:** Tianjin Key Laboratory of Retinal Functions and Diseases, Tianjin Branch of National Clinical Research Center for Ocular Disease, Eye Institute and School of Optometry, Tianjin Medical University Eye Hospital, Tianjin, 300384 China

**Keywords:** Orthokeratology, DISC, Axial elongation, Myopia control

## Abstract

**Background:**

To compare axial elongation in 8–11-year-old myopes wearing orthokeratology (OK) lenses with different back optic zone diameters (BOZD), defocus incorporated soft contact (DISC) lenses, and single-vision soft contact lenses (SCLs).

**Methods:**

A total of 122 children (aged 8–11 years) with spherical equivalent refraction (SER) between − 1.00 D and − 4.00 D were enrolled in this prospective study and randomly assigned to four groups: 5.0 mm-BOZD OK, 6.2 mm-BOZD OK, DISC, and single-vision SCLs. Children in each group were further divided into subgroups stratified by the average baseline SER: low myopic eyes (SER: − 1.00 D to − 2.50 D) and moderate myopic eyes (SER: − 2.50 D and over). Axial length (AL) was measured at baseline and after one year.

**Results:**

The 5.0 mm-BOZD OK, 6.2 mm-BOZD OK, and DISC groups exhibited significantly slower AL elongation than the SCL group. The proportion of slow progressors (AL elongation ≤ 0.18 mm/year) in the first three groups was 42%, 23%, and 29%, respectively. Furthermore, one-year AL elongation was significantly smaller in the 5.0 mm-BOZD OK group compared with the 6.2 mm-BOZD OK group. Regardless of SER, children in the 5.0 mm-BOZD OK and DISC groups showed comparably slower AL elongation than those in the SCL group. However, fitting with 6.2 mm-BOZD OK lenses significantly retarded AL elongation in moderate myopic eyes, but not in low myopic eyes.

**Conclusions:**

Overall**,** 5.0 mm-BOZD OK lenses, 6.2 mm-BOZD OK lenses, and DISC lenses were effective in retarding AL elongation in 8–11-year-old myopes compared with single-vision SCLs, but for children with SER less than − 2.50 D, fitting with 5.0 mm-BOZD OK lenses and DISC lenses yielded better myopia control efficacy compared to wearing single-vision SCLs or 6.2 mm-BOZD OK lenses.

## Background

Due to its growing prevalence and the increased risk of related ocular complications such as myopic maculopathy and high myopia-associated optic neuropathy, myopia has become a global public health issue [1, 2]. Its global prevalence is projected to reach 49.8% by 2050, with the increase being particularly evident in East and Southeast Asia [3]. Since myopia usually manifests at a young age (7–10 years) [4], delivering interventions in childhood is beneficial for myopia control.

Orthokeratology (OK) lenses and soft contact lenses (SCLs) with concentric ring bifocal or peripheral add multifocal designs are two well-accepted clinical treatments for correcting refractive error and retarding myopia progression [2, 5]. Relative myopic peripheral defocus is considered one of the mechanisms by which OK lenses and bifocal or multifocal defocus SCLs slow myopia progression [6, 7]. The inhibitory effect on axial length (AL) elongation in children with myopia reportedly varies from 30% to 60% for OK treatment over a one-year follow-up [8–14], and from 38% to 87% for bifocal or multifocal defocus SCLs after one year of wearing [15–20]. In addition to the differences in subject groups, there are distinctive differences in lens designs between the contact lenses used in different studies, which may contribute to variability in the rates of myopic progression.

Recent studies have focused on modifying the design of OK lenses or myopia-controlling SCLs to improve the myopia control effect [21–24]. We previously reported that OK lenses with a smaller back optic zone diameter (BOZD) showed increased myopia control efficacy among 8–11-year-old children compared with larger BOZD OK lenses [22]. Dual-focus SCLs with different optic designs and additions, such as the defocus incorporated soft contact (DISC) lens and MiSight® lens, effectively control myopia and are widely applied in clinical settings [16, 25]. Of these, daily disposable DISC lenses, with a concentric ring design comprising a central correction zone and a series of alternating defocusing (+ 2.50 D addition) and correction zones extending toward the periphery, effectively retarded AL elongation in Hong Kong schoolchildren compared with single-vision SCLs during a two-year follow-up [16]. MiSight® lenses with peripheral concentric + 2.00 D add rings significantly slowed AL elongation and the myopia refraction increase, when compared to the control SCLs over three years [26]. As indicated above, both optimized-design OK lenses and dual-focus SCLs are effective strategies for controlling myopia progression. Although Turnbull et al. [27] found no significant differences in the myopia control efficacy of corneal refractive therapy OK lenses (CRT™, Paragon Vision Sciences, Mesa, AZ, USA) and dual-focus SCLs (MiSight® lenses or custom-made dual-focus lenses with concentric + 2.00 D add zones), the relative effectiveness of OK lenses compared with DISC lenses has not been specifically examined.

Children (8–11 years old) with myopia present with relatively fast axial growth, thereby requiring effective myopia control interventions [28]. To that end, the present study aimed to compare the myopia control efficacy of 6.2 mm-BOZD OK lenses, 5.0 mm-BOZD OK lenses, and DISC lenses in 8–11-year-old children to suggest insights for clinicians in personalizing myopia control measures.

## Methods

### Participants

A total of 141 participants were enrolled in this prospective study. This study was approved by the Ethics Committee of Tianjin Medical University Eye Hospital (Permit Number: 202005), and all procedures complied with the tenets of the Declaration of Helsinki. All examinations were conducted after the participants and their guardians fully understood the study details and signed the informed consent forms. The inclusion criteria were as follows: age between 8–11 years, cycloplegic spherical power between − 1.00 to − 4.00 D, with-the-rule astigmatism ≤  − 0.75 D, and best-corrected visual acuity no worse than 20/20. Individuals with strabismus or ocular surface disease, a history of ocular surgery, or a history of contact lens wear in the past 30 days were excluded.

### Lens fitting

Two types of OK lenses, a double reservoir lens (DRL®, Precilens, Creteil, France) with a 5.0 mm-BOZD and Euclid lens (Euclid Systems Corporation, Herndon, VA) with a 6.2 mm-BOZD, designed to alter the anterior corneal curvature during overnight wear, were used. The related detailed information is listed in Table 1. All OK lenses had a spherical design and were fitted to both eyes according to the manufacturer’s guidelines. Lenses were ordered with over-correction targeted at + 0.75 D. Participants were instructed to wear the lenses overnight and for at least 8 h per night and 6 nights per week to maintain good daytime vision after removing lenses. The OK lens prescriptions were changed only when the unaided monocular visual acuity was worse than 20/25 or when significant lens decentration was observed. Follow-up examinations were conducted at 1 day, 1 week, and 1 month after the initial lens fitting and at least once every three months thereafter.

**Table 1:** Detailed information about OK lenses and SCLs

OK lenses	DRL	Euclid
Design	Base curve, reverse curve, alignment curve and peripheral curve	Base curve, reverse curve, alignment curve, and peripheral curve
TD	8.0–12.6 mm	9.6–11.6 mm
BOZD	5.0 mm	6.2 mm
RCW	0.8–1.6 mm	0.5–0.6 mm
ACW	0.3–1.1 mm	0.9–1.6 mm
Central thickness	0.20–0.25 mm	0.20–0.32 mm
Dk	100 × 10^–11^(cm^2^/s) [mlO_2_/(ml·mm Hg)]	87 × 10^–11^(cm^2^/s)·[mlO_2_/(ml·mm Hg)]
Material	Boston XO	Boston Equalens II

SCLs	DISC	Single-vision SCLs
Design	Concentric-ring and dual-focus	Single-vision
Base curve	8.6 mm	8.6 mm
Defocus addition	+ 2.50 D	None
TD	14.2 mm	14.2 mm
Central optical zone	3.0 mm	8.0 mm
Water content	38%	38%
Central thickness	0.1 mm	0.1 mm
Dk	8.4 × 10^–11^ (cm^2^/s) ·[mlO_2_/(ml·mm Hg)]	8.4 × 10^–11^ (cm^2^/s)·[mlO_2_/(ml·mm Hg)]
Material	hydroxyethyl methacrylate	hydroxyethyl methacrylate

*OK* = orthokeratology; *DRL* = double reservoir lens; *DISC* = defocus incorporated soft contact; *SCL* = soft contact lens; *TD* = total lens diameter; *BOZD* = back optic zone diameter; *Dk* = oxygen permeability; *RCW* = reverse curve width; *ACW* = alignment curve width

Daily disposable single-vision SCLs and DISC lenses with concentric ring design were produced by St. Shine Optical Co., Ltd. (Taiwan, China), and made from hydroxyethyl methacrylate, with 38% water content, a diameter of 14.2 mm, and a base curve of 8.6 mm (Table 1). The DISC lenses were designed to have a spherical distance power at the central optical zone of 3.0 mm diameter and a series of alternating defocusing (+ 2.50 D add) and correction concentric rings with a width of 0.25 mm and having a proportion of 50:50 [16]. Single-vision SCLs had an 8.0 mm-optical zone diameter. Participants were asked to wear the SCLs during the daytime for at least 6 days per week and 8 h per day. If the monocular corrected visual acuity was less than 20/25, or the spherical over-refraction achieved − 0.50 D, the SCL prescription had to be modified. Follow-up examinations were performed at least once every three months after commencing lens wear.

All participants underwent a comprehensive ocular examination assessment, including uncorrected and corrected distance visual acuity, manifest refraction, and slit-lamp examination at each follow-up visit.

### Sample size

The sample size calculation was based on the number of participants needed to detect differences in AL elongation of at least 0.15 mm/year among groups [29], with a power of 80% and a significance level of alpha = 0.05. For these calculations, we assumed a measurement SD of 0.15 mm [12, 30]. Thus, a minimum sample size of 19 was required for each group. Taking into account 20% loss to follow-up, about 24 participants should be recruited in each group.

### Groups

Participants were randomly assigned to either the 5.0 mm-BOZD OK group, 6.2 mm-BOZD OK group, DISC group, or single-vision SCL group. The randomization scheme for the study was generated using a commercial spreadsheet generator (Excel; Microsoft, Redmond, WA), and the treatment assignment was sealed in opaque envelopes. In order to ensure allocation concealment, the envelope was handed directly to the subjects at randomization.

The mean baseline spherical equivalent (SER, − 2.50 D) of the study cohort was selected as a cut-off value. On this basis, participants in the 5.0 mm-BOZD OK group, 6.2 mm-BOZD OK group, DISC group, and single-vision SCL group were further classified into low myopic (SER: − 1.00 D to − 2.50 D) and moderate myopic (SER: − 2.50 D and over) subgroups.

Based on the outcomes of one-year axial elongation, participants with slow myopic progression were screened from the 5.0 mm-BOZD OK, 6.2 mm-BOZD OK, DISC, and single-vision SCL groups for further analysis. Those with AL elongation ≤ 0.18 mm/year [9, 14] were regarded as slow progressors. The proportions of slow progressors in the four groups were calculated and compared.

### Refraction

At baseline, the cycloplegic refraction of all participants was examined. Cycloplegia was conducted by putting four drops of 5 mg/ml tropicamide eye drops instilled 5 min apart in each eye. At least 20 min after the last eye drop, complete cycloplegia was evaluated by an absence of light reflex and a dilated pupil at least 6.0 mm in diameter, and then subjective refraction was performed by the same optometrist. SER was calculated as the sum of the sphere plus 0.5 cylinder power. At the 12-month follow-up visit, the cycloplegic refraction was performed three hours after the removal of lenses only in participants wearing DISC or single-vision SCL.

### AL measurement

AL was measured at baseline and the 12-month follow-up visit (three hours after the removal of lenses) using noncontact optical biometry (Lenstar LS900 Haag-Streit, Koeniz, Switzerland), and the difference between the two-time points was recorded. At each visit, three AL measurements were recorded. If the between-measurement difference was greater than 0.02 mm [31], the three measurements were repeated until the between-measurement difference was less than 0.02 mm; these were then averaged as the representative value for analysis.

### Corneal topography

**Fig. 1:**
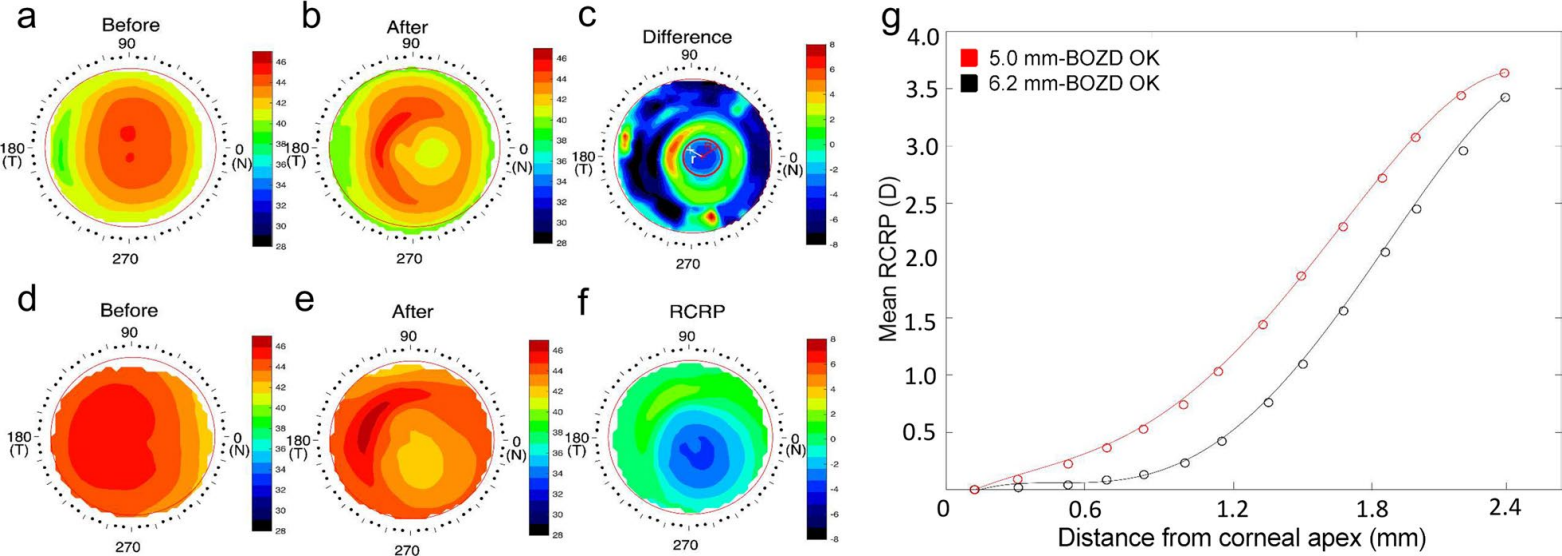
Methods to determine the treatment zone size, the treatment zone decentration and the RCRP for OK wearers. **a** Tangential curvature map at baseline; **b** Tangential curvature map at the 12-month visit; **c** Difference map used to determine the treatment zone size and decentration. The red circle represents the boundary of the treatment zone. The red cross indicates the center of the circle, and R is the circle radius used for calculating treatment zone diameter. The white cross represents the corneal apex, and r represents the distance of treatment zone decentration. **d** Axial power map at baseline; **e** Axial power map at the 12-month visit; **f** The RCRP map; **g** Representative examples from individual participants of mean RCRP profiles within the average pupillary diameter (4.80 mm) in the 5.0 mm-BOZD OK group and 6.2 mm-BOZD OK group. BOZD, back optic zone diameter; OK, orthokeratology; RCRP, relative corneal refractive power

Corneal topography was captured using TMS-4 (Tomey, Nagoya, Japan) at baseline for all participants. During the follow-up visit, only OK wearers underwent corneal topography examination. Three images, which provided an optimum index value according to the manufacturer’s recommendations, were saved and used for further corneal topography analysis in OK-wearing participants. The treatment zone size and decentration were calculated according to our previous studies [22, 32]. In detail, a difference map was obtained by subtracting the tangential curvature map collected at the one-year visit (Fig. 1b) from the baseline map (Fig. 1a). The area containing locations reduced by > 0.00 D was considered as the treatment zone, and its boundary was fitted to a circle using a custom MATLAB function (MathWorks, Natick, WA, USA) (Fig. 1c). The diameter of the fitted circle was defined as the treatment zone size, and the distance between the circle’s center (red cross) and the geometric center of the cornea (white cross) was defined as the treatment zone decentration.

Axial power maps were used to analyze the relative corneal refractive power (RCRP) [22]. With the corneal apex as the center, each axial power map contained 31 rings with a ring interval of 0.162 mm and 256 data points for each ring. The RCRP map (Fig. 1f) for OK wearers was derived by subtracting the apical corneal refractive power from the power of each point on the post-treatment axial power map (Fig. 1e).The pupil diameter was obtained from the topographic images captured under ambient mesopic room illumination [33]. Since the mean pupil diameter of children included in OK groups was 4.80 mm (± 0.72 mm), the RCRP values of data points in the first 14 rings (the central area with a diameter of 4.80 mm) were averaged along each ring to derive the mean RCRP value, and a quadratic curve was fitted using the 14 mean values for OK groups. Representative examples from individual participants of mean RCRP profiles within the average pupillary diameter (4.80 mm) in the 5.0 mm-BOZD OK group and 6.2 mm-BOZD OK group are shown in Fig. 1g. The sum of the first 14 mean values on the RCRP profile within 4.80 mm in diameter (Sum4.8) was calculated to reflect the OK lens-induced corneal power shift summed within the central pupillary area. The number of all data points on the RCRP map within the individual pupillary diameter and the number of data points with a refraction power of more than 0.00 D on the RCRP map within the individual pupillary diameter was calculated, and the ratio of the latter to the former was defined as the percentage of defocus zone within the pupil area.

### Statistical analysis

Data from the right eye were used for statistical analysis. The normality of the data was tested using the Shapiro–Wilk test. Differences between the 5.0 mm-BOZD OK group and the 6.2 mm-BOZD OK group were tested using the unpaired t-test for quantitative data and the Mann–Whitney U test for non-parametric data. When normality was not rejected, comparisons among the four groups were performed using the one-way analysis of variance (ANOVA). Non-parametric data from the four groups were compared using the Kruskal–Wallis H test. Post-hoc comparisons using Bonferroni corrections were performed for significant outcomes. Chi-square test for categorical variables using 2 × C contingency table was performed to compare the male/female ratio (M/F ratio) difference among four groups, and compare the proportions of slow progressors (AL elongation ≤ 0.18 mm/year) [9, 14] among the 5.0 mm-BOZD OK, 6.2 mm-BOZD OK, and DISC groups. Post-hoc comparisons using Bonferroni corrections were performed for significant outcomes. All analyses were performed using SPSS software version 25.0 (IBM Corp., Armonk, NY, USA). Results with *P* < 0.05 were considered statistically significant.

## Results

**Fig. 2:**
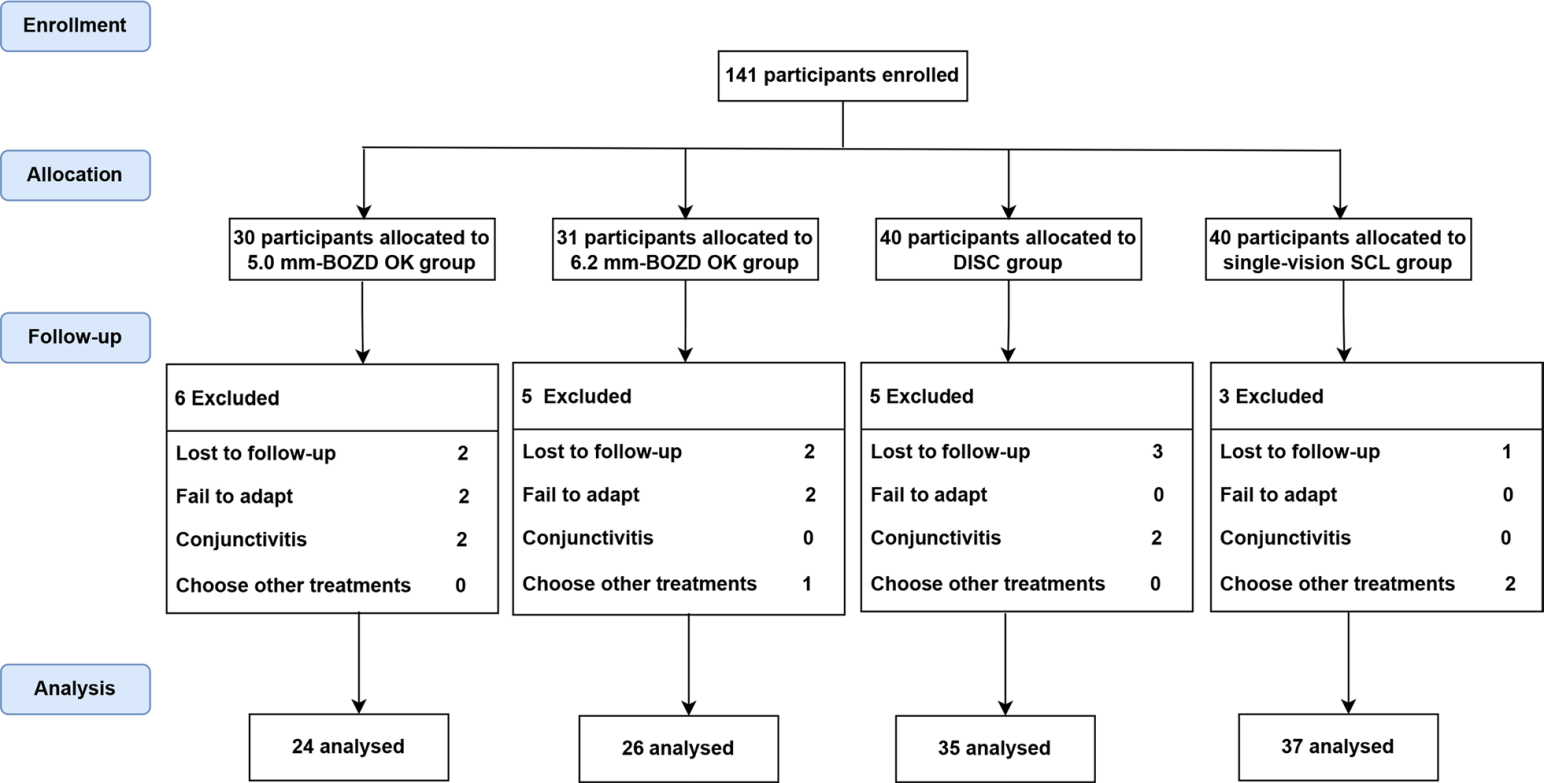
Flow diagram of study progress in the 5.0 mm-BOZD OK, 6.2 mm-BOZD OK, DISC and SCL groups. BOZD, back optic zone diameter; OK, orthokeratology; DISC, defocus incorporated soft contact; SCL, soft contact lens

A total of 122 children (86.5%) completed all measurements during the one-year follow-ups. Nineteen children could not continue with the study due to various reasons. Eight children were lost to follow-up. Four children failed to adapt to lens wear. Four children were excluded due to conjunctivitis. Three children dropped out due to their parents' preference to choose other myopia control treatments. Hence, their data were excluded from the final analysis (Fig. 2). At baseline, no significant differences were observed among the four groups in terms of age, sex distribution, SER, or AL (all *P* > 0.05, Table 2).

**Table 2:** Baseline information for participants in four groups

Parameter	5.0 mm-BOZD OK (n = 24)	6.2 mm-BOZD OK (n = 26)	DISC (n=35)	SCL (n=37)	*P* value
Age (years)	9 [8, 11]	9.50 [8, 11]	10 [8, 11]	10 [8, 11]	0.88^#^
Sex (M/F)	12/12	12/14	18/17	18/19	0.98^&^
SER (D)	− 2.38 [− 4.00, − 1.25]	− 2.25 [− 4.00, − 1.00]	− 2.50 [− 4.00, − 1.00]	− 2.25 [− 4.00, − 1.00]	0.94^#^
AL (mm)	24.67 ± 0.60	24.63 ± 0.78	24.40 ± 0.71	24.39 ± 0.57	0.24^*^

*BOZD* = back optic zone diameter; *OK* = orthokeratology; *DISC* = defocus incorporated soft contact; *SCL* = soft contact lens; *SER* = spherical equivalent refraction; *AL* = axial length. Data are expressed as the mean ± SD or median [range]

^#^Kruskal–Wallis H test

^&^Chi-square test

^*^One-way ANOVA

**Fig. 3:**
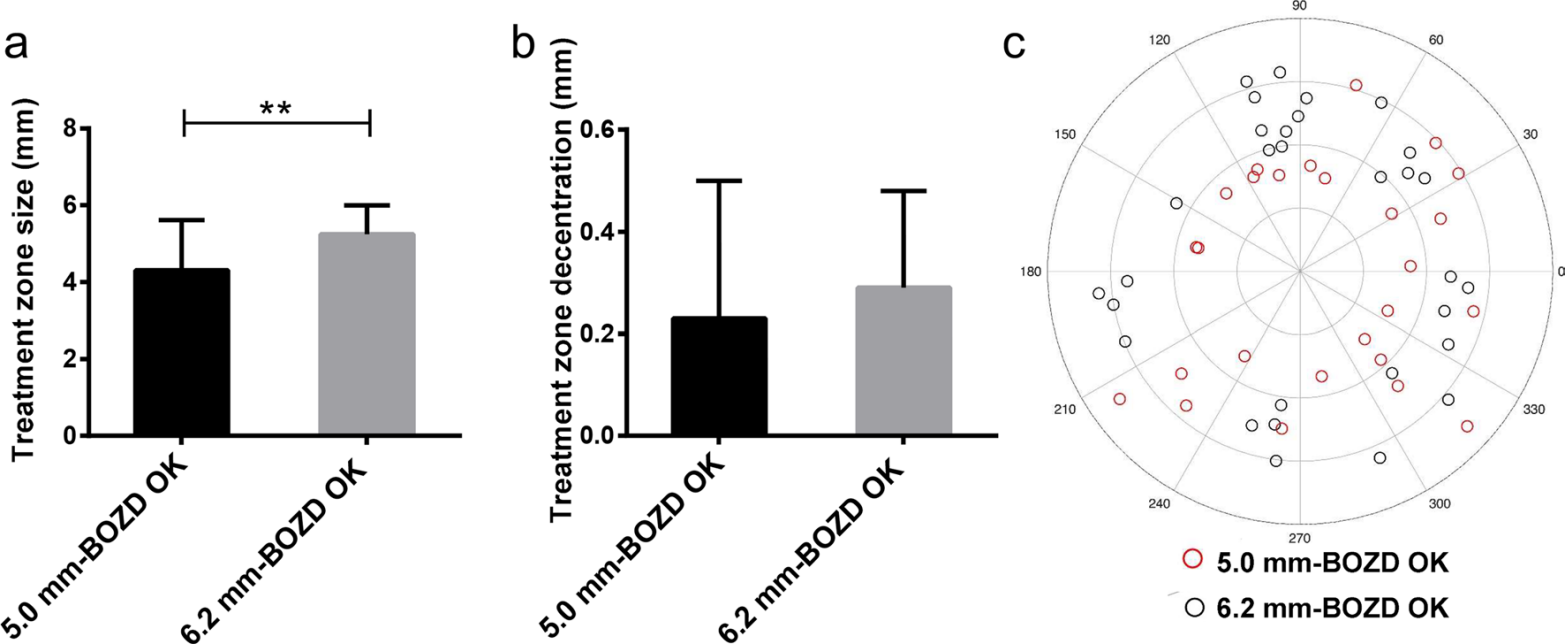
Treatment zone size (**a**), treatment zone decentration distance (**b**) and polar plot displaying the treatment zone decentration direction (**c**) in the 5.0 mm-BOZD OK and the 6.2 mm-BOZD OK groups. The range between 0° and 360° is similar to the meridian degree set on a corneal topography map, and the small circle represents the center of the treatment zone (**c**). Error bars represent the standard deviation (**a** and **b**). BOZD, back optic zone diameter; OK, orthokeratology. **P < 0.01

**Fig. 4:**
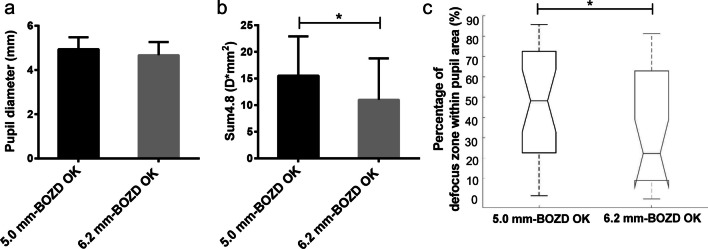
The pupil size (**a**), the sum value of the RCRP within 4.80 mm diameter (Sum4.8) (**b**) and the percentage of defocus zone within the pupil area (**c**) in the 5.0 mm-BOZD OK and the 6.2 mm-BOZD OK groups. The boxplots in (**c**) extend from the lower to upper quartile values of the data, with a line at the median. The whiskers extend from the box to show the range of the data. RCRP, relative corneal refractive power; BOZD, back optic zone diameter; OK, orthokeratology. Error bars represent the standard deviation (**a** and **b**), or range (**c**). *P < 0.05

The treatment zone size in the 5.0 mm-BOZD OK group (4.30 ± 1.31 mm in diameter) was significantly smaller than that in the 6.2 mm-BOZD OK group (5.24 ± 0.75 mm in diameter; *P* < 0.01, Fig. 3a). However, the distance of treatment zone decentration was not significantly different between the two types of lenses (0.23 ± 0.27 mm for 5.0 mm-BOZD OK group vs. 0.29 ± 0.19 mm for 6.2 mm-BOZD OK group; *P* > 0.05, Fig. 3b), and the decentration direction was similar between two groups (179.82 ± 107.72 degree for 5.0 mm-BOZD OK group vs. 174.85 ± 112.53 degree for 6.2 mm-BOZD OK group; *P* > 0.05, Fig. 3c). The pupil diameters of the children in the 5.0 mm-BOZD OK group and the 6.2 mm-BOZD OK group were 4.94 ± 0.54 mm and 4.67 ± 0.60 mm, respectively, and no significant differences were found between groups (*P* > 0.05, Fig. 4a). The pupil diameter of all OK wearers was 4.80 ± 0.72 mm. Representative examples from individual participants of mean RCRP profiles within the average pupillary diameter (4.80 mm) in the 5.0 mm-BOZD OK group and 6.2 mm-BOZD OK group are shown in Fig. 1g. Children wearing the 5.0 mm-BOZD OK lenses had a significantly larger RCRP sum within the 4.80-mm diameter zone (Sum4.8) than those wearing 6.2 mm-BOZD OK lenses (15.54 ± 7.36 D*mm^2^ for 5.0 mm-BOZD OK group vs. 11.02 ± 7.77 D*mm^2^ for 6.2 mm-BOZD OK group; *P* < 0.05, Fig. 4b). The 5.0 mm-BOZD OK group showed a significantly larger percentage of defocus zone within the pupil area than the 6.2 mm-BOZD OK group, 48.22% (range from 1.53% to 85.73%) vs. 22.38% (range from 0% to 81.19%) (*P* < 0.05, Fig. 4c).

**Fig. 5:**
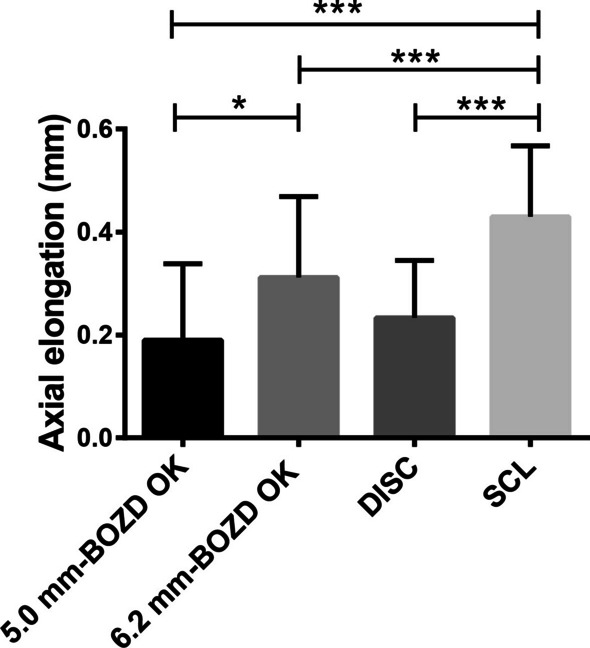
Axial elongation over one year in the 5.0 mm-BOZD OK, 6.2 mm-BOZD OK, DISC and SCL groups. BOZD, back optic zone diameter; OK, orthokeratology; DISC, defocus incorporated soft contact; SCL, soft contact lens. Error bars represent the standard deviation. *P < 0.05, ***P < 0.001

There were significant differences in the one-year AL elongation among the four groups (*P* < 0.01; Fig. 5). Subsequent Bonferroni-adjusted post-hoc comparisons indicated that the one-year AL elongation in the 5.0 mm-BOZD OK group, 6.2 mm-BOZD OK group, and DISC group were 0.19 ± 0.14 mm, 0.31 ± 0.15 mm, and 0.23 ± 0.11 mm, respectively, which were all significantly smaller than that (0.43 ± 0.13 mm) in the SCL group (all *P* < 0.01). Compared with the 6.2 mm-BOZD OK group, children in the 5.0 mm-BOZD OK group presented significantly slower AL elongation (*P* < 0.05). However, no significant difference in AL elongation was observed between the 5.0 mm-BOZD OK and the DISC groups (*P* > 0.99). Furthermore, the mean AL elongation was smaller in the DISC group than in the 6.2 mm-BOZD OK group, but the difference was not statistically significant (*P* > 0.05).

**Fig. 6:**
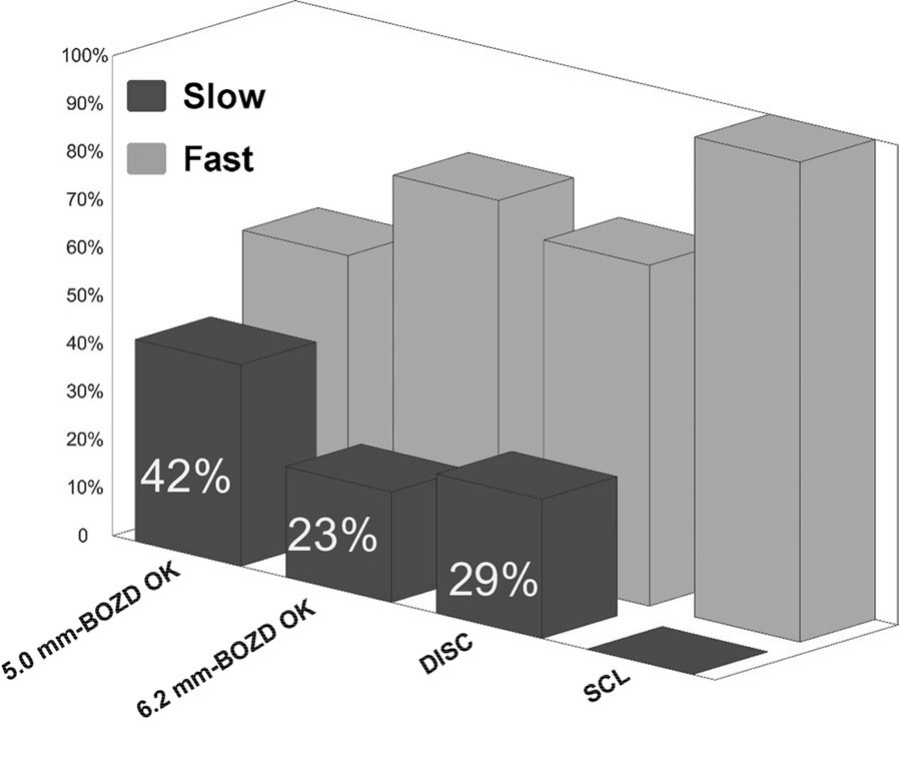
Percentage of subjects demonstrating slow (≤ 0.18 mm/year) and fast (> 0.18 mm/year) myopic progression in the 5.0 mm-BOZD OK, 6.2 mm-BOZD OK, DISC and SCL groups. BOZD, back optic zone diameter; OK, orthokeratology; DISC, defocus incorporated soft contact; SCL, soft contact lens

Figure 6 shows the percentage of children with different myopic progression rates. Children with AL elongation less than 0.18 mm/year were regarded as slow progressors [9, 14]. Slow progression was not observed in the SCL group. There were significant differences in the proportions of slow progressors among the other three groups (χ^2^ = 8.77, *P* < 0.05). Bonferroni-adjusted post-hoc comparisons between groups showed that the 5.0 mm-BOZD OK group had a greater number of slow progressors (42%) compared with the 6.2 mm-BOZD OK group (23%; *P* < 0.05). There was no significant difference in the proportions of slow progressors between the 5.0 mm-BOZD OK group and DISC group as well as between the 6.2 mm-BOZD OK group and DISC group (*P* > 0.05). Compared with the SCL group, AL elongation was reduced by 55.81% in the 5.0 mm-BOZD OK group, 27.90% in the 6.2 mm-BOZD OK group and 46.51% in the DISC group (Table 3).

**Table 3:** Percent reduction in AL elongation for the 5.0 mm-BOZD OK, 6.2 mm-BOZD OK and DISC groups versus the SCL group

Parameter	5.0 mm-BOZD OK (%)	6.2 mm-BOZD OK (%)	DISC (%)
Overall	55.81	27.90	46.51
Low myopic groups (SER: − 1.00 D to − 2.50 D)	47.61	11.90	42.86
Moderate myopic groups (SER: − 2.50 D and over)	65.12	44.19	48.84

*BOZD* = back optic zone diameter; *OK* = orthokeratology; *DISC* = defocus incorporated soft contact; *SCL* = soft contact lens; *SER* = spherical equivalent refraction

The myopic control effect may be affected by the baseline SER [13, 34]. Given this, subjects in different groups were further stratified into low and moderate myopic eye groups based on an average SER of − 2.50 D. Baseline information in the subgroups is shown in Table 4; there were no significant differences in the age, sex distribution, SER, and AL among the four subgroups in either the low or moderate myopic eye groups (all *P* > 0.05, Table 4).

**Table 4:** Baseline information for participants in low myopic (SER: − 1.00 D to − 2.50 D) and moderate myopic (SER: − 2.50 D and over) groups

Low myopic groups (n=63)	5.0 mm-BOZD OK (n = 12)	6.2 mm-BOZD OK (n = 14)	DISC (n=17)	SCL (n=20)	*P* value
Age (years)	9 [8, 11]	9[8, 11]	10 [8, 11]	9 [8, 11]	0.43^#^
Sex (M/F)	5/7	7/7	8/9	9/11	0.98^&^
SER (D)	− 1.77 ± 0.37	− 1.80 ± 0.41	− 1.65 ± 0.36	− 1.91 ± 0.27	0.19^*^
AL (mm)	24.32 [23.40, 25.30]	24.14 [23.28, 24.79]	24.19 [23.03, 25.18]	24.3[23.14, 24.80]	0.62^#^

Moderate myopic groups (n = 59)	5.0 mm-BOZD OK (n = 12)	6.2 mm-BOZD OK (n = 12)	DISC (n = 18)	SCL (n = 17)	*P* value
Age (years)	10 [8, 11]	10 [8, 11]	10 [8, 11]	10 [8, 11]	0.94^#^
Sex (M/F)	7/5	5/7	10/8	9/8	0.85^&^
SER (D)	− 3.25 [− 4.00, − 2.50]	− 3.56 [− 4.00, − 2.50]	− 3.00 [− 4.00, − 2.50]	− 3.25 [− 4.00, − 2.50]	0.51^#^
AL (mm)	24.88 [24.49, 25.96]	25.00 [24.27, 26.17]	24.78 [23.83, 25.79]	24.60 [23.31, 25.92]	0.09^#^

*SER* = spherical equivalent refraction; *BOZD* = back optic zone diameter; *OK* = orthokeratology; *DISC* = defocus incorporated soft contact; *SCL*= soft contact lens; *AL* = axial length. Data are expressed as the mean ± SD or median [range]

^#^Kruskal–Wallis H test

^&^Chi-square test

^*^One-way ANOVA

**Fig. 7:**
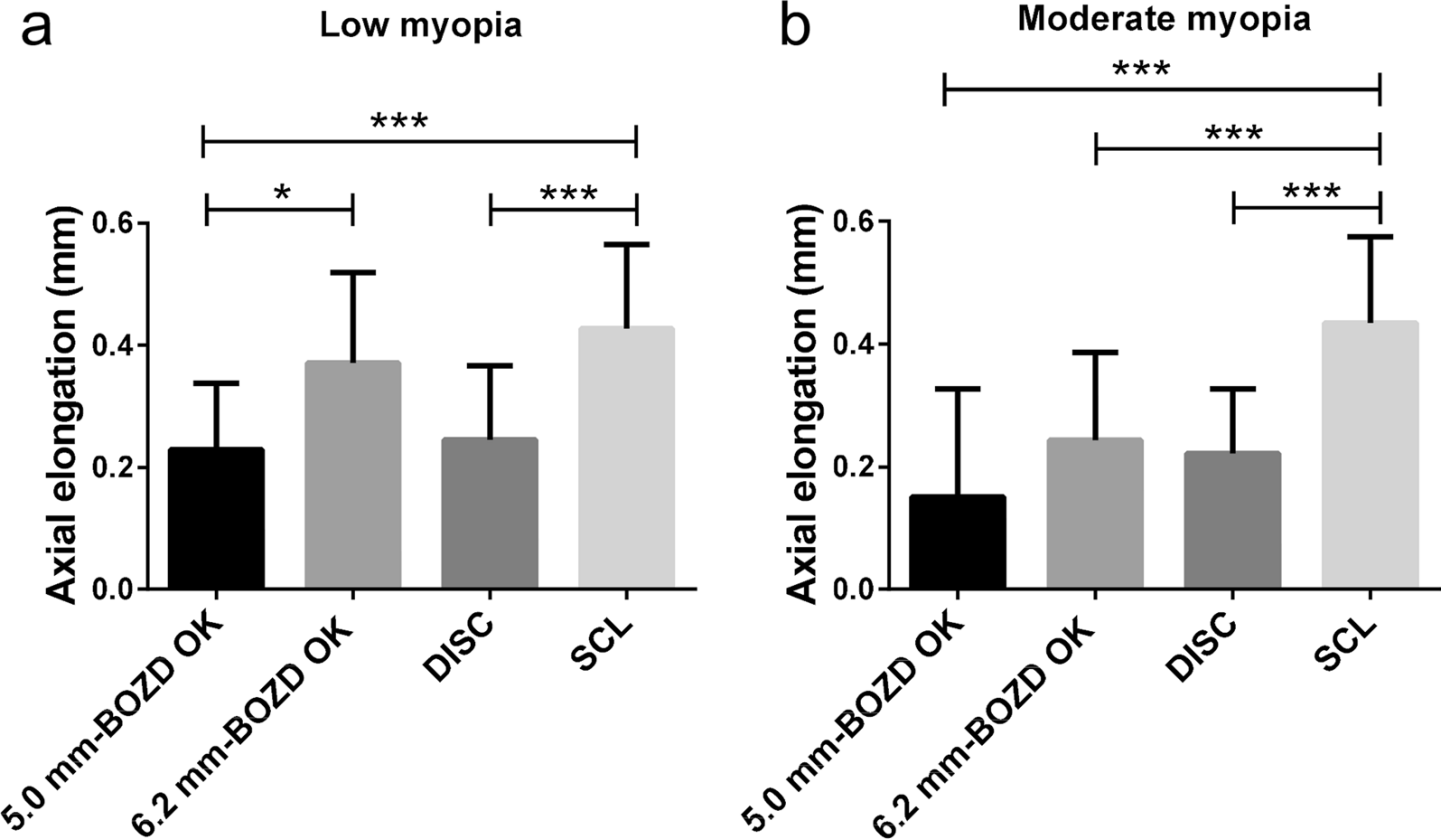
Axial elongation over one year in low myopic (SER: − 1.00 D to − 2.50 D) and moderate myopic (SER: − 2.50 D and over) groups wearing 5.0 mm-BOZD OK, 6.2 mm-BOZD OK, DISC, and SCL. SER, spherical equivalent refraction; BOZD, back optic zone diameter; OK, orthokeratology; DISC, defocus incorporated soft contact; SCL, soft contact lens. Error bars represent the standard deviation. *P < 0.05, ***P < 0.001

As shown in Fig. 7a, for eyes with low myopia, the AL elongation in the 5.0 mm-BOZD OK group (0.22 ± 0.10 mm) and DISC group (0.24 ± 0.12 mm) were markedly smaller than that (0.42 ± 0.13 mm) in the SCL group (both *P* < 0.01). However, the AL elongation in the 6.2 mm-BOZD OK and SCL groups was comparable (*P* > 0.99). AL elongation was significantly slower in the 5.0 mm-BOZD OK group than in the 6.2 mm-BOZD OK group (*P* < 0.05). Although per year AL elongation in the 6.2 mm-BOZD OK group (0.37 ± 0.14 mm) was slightly larger than that in the DISC group (0.24 ± 0.12 mm), the difference was not statistically significant (*P* > 0.05). In comparison with the SCL group, AL elongation was reduced by 47.61% in the 5.0 mm-BOZD OK group, 11.90% in the 6.2 mm-BOZD OK group and 42.86% in the DISC group (Table 3).

For moderate myopic eyes, AL elongation was significantly slower in the 5.0 mm-BOZD OK (0.15 ± 0.17 mm), 6.2 mm-BOZD OK (0.24 ± 0.14 mm), and DISC groups (0.22 ± 0.10 mm) compared to the SCL group (0.43 ± 0.14 mm, all *P* < 0.01, Fig. 7b). Among the 5.0 mm-BOZD OK, 6.2 mm-BOZD OK, and SCL groups, the differences in AL elongation were not statistically significant (all *P* > 0.05, Fig. 7b). Percentage reductions in AL elongation in the 5.0 mm-BOZD OK group, 6.2 mm-BOZD OK group, and DISC group versus the SCL group were 65.12%, 44.19% and 48.84%, respectively (Table 3).

## Discussion

In the present study, we demonstrated that compared to single-vision SCLs, 5.0 mm-BOZD OK lenses, 6.2 mm-BOZD OK lenses, and DISC lenses were effective in slowing AL elongation in 8–11-year-old children with myopia. The 5.0 mm-BOZD OK group had the largest proportion of slow progressors (42%; AL increase ≤ 0.18 mm/year). Notably, for low myopia with SER less than − 2.50 D, 5.0 mm-BOZD OK lenses and DISC lenses yielded better myopic control than single-vision SCLs or 6.2 mm-BOZD OK lenses. These findings provide guidelines for clinicians in choosing effective myopia control strategies for young children with low myopia.

Both overnight OK lenses and daytime myopia controlling SCLs are clinically effective for controlling AL elongation, but display diverse efficacies in different participant groups [35, 36]. For example, we previously reported that 8–11-year-old children with myopia showed a relatively fast AL elongation after wearing traditional 6.2 mm-BOZD OK lenses compared to children older than 11 years [28]. As for OK lenses, studies have shown that different brands of traditional vision shaping treatment (VST) OK lenses with typically 6.0 mm-BOZD and 4-curve lens design had similar efficacy in slowing axial elongation [37, 38], and corneal refractive therapy (CRT™) OK lenses with 6.0 mm-BOZD and 3-curve lens design demonstrated a weaker myopia control effect when compared with traditional VST lenses during 1 to 1.5 year-follow-up [37, 39]. With respect to myopia controlling SCLs, alternating bifocal design or progressive multifocal design are widely used in the clinic, but concentric ring bifocal SCLs are reportedly better at slowing AL elongation than peripheral multifocal SCLs [36].

Recent studies have aimed to explore new optical designs for OK lenses or SCLs to enhance myopia control efficacy [22, 36, 40]. Decreasing the BOZD design in OK lenses has better controlled AL elongation in our previous study [22] as well as other studies [21, 41, 42]. For instance, Pauné et al. found a 0.06 mm/year less AL growth in Caucasian children with 4.7 or 5.0 mm-BOZD DRL lenses compared with those wearing lenses with a BOZD ranging from 5.6 to 6.6 mm [21]. Guo et al. reported that AL elongation in Chinese children wearing 5.0 mm-BOZD KATT lenses (KATT MC, Precision Technology Services, ptsoptics.com) decreased by 0.13 mm/year compared with those wearing 6.0 mm-BOZD lenses [41]. Bifocal SCLs with different sizes of central distance correction zones and various designs of concentric defocus rings have also been developed [23]. For example, MiSight® lenses, containing a 3.36 mm central correction area and two peripheral concentric + 2.00 D add rings [43], and DISC lenses, comprising a 3.0 mm central correction zone and five peripheral concentric + 2.50 D defocus rings [16] have been proven to be effective in slowing myopia progression in children compared with control groups. However, the myopia control effectiveness of smaller BOZD OK lenses and bifocal SCLs such as DISC lenses has not been compared in 8–11-year-old children whose myopia progressed faster than that of older children.

In this study, we compared the efficacy of 5.0 mm-BOZD OK lenses, 6.2 mm-BOZD OK lenses, and DISC lenses (bifocal SCLs with concentric + 2.50 D add zones) for myopia control in 8–11-year-old children with myopia and found that these three lenses effectively retarded AL elongation within 12 months when compared with single-vision SCLs, and showed AL growth reduction efficacies of 56%, 28%, and 47%, respectively. Expectedly, AL elongation was significantly slowed down more in the 5.0 mm-BOZD OK group than in the 6.2 mm-BOZD OK group, as reported in previous studies [21, 22, 41]. The AL elongation control efficacy of DISC lenses in the present study was similar to that reported for Hong Kong schoolchildren by Lam et al. [16], who found that myopia in children wearing DISC lenses for five or more hours/day progressed 46% less than those fitting with single-vision SCLs. Although a 0.08 mm/year lower AL increase was observed in those wearing DISC lenses compared to those wearing 6.2 mm-BOZD OK lenses, the difference was not statistically significant. This finding was in accordance with those reported in a previous study, which showed no difference in per annum AL elongation inhibition by traditional CRT OK lenses and dual-focus SCLs in pediatric patients with myopia (average age: 12 years) [27]. However, owing to the designs of OK lenses and dual-focus SCLs being completely different from those in the present study, the results warrant further verification. In the present study, 5.0 mm-BOZD OK lenses showed a similar retardation effect on AL elongation as compared with DISC lenses. Given the relatively high control efficacies of the two lenses, fitting with 5.0 mm-BOZD OK lenses or DISC lenses may be the first recommendation for myopia control in 8–11-year-old children with myopia.

The average AL elongation per year in Chinese children varies with age, ranging from 0.16 mm to 0.41 mm in children aged 8 to 11 years old [44]. Previous studies have considered 6–11-year-old Chinese children with annual AL elongation lower than 0.18 mm as slow progressors [9, 14]. In the present study, the proportion of slow progressors in the 5.0 mm-BOZD OK group was 42%, which was higher than that in the 6.2 mm-BOZD OK group (23%) and DISC group (29%). Nevertheless, He et al. found that the percentage of slow progressors was 41.8% in the 7–11-year-old Chinese children wearing 6.2 mm-BOZD OK lenses (Lucid, Korea) for one year [14], which was inconsistent with our result observed in the 6.2 mm-BOZD OK group. Considering that the proportions of slow progressors in the control group differ greatly between He et al.’s and our study (11.5% in the former, and 0% in the latter), one possible explanation is that the axial growth of the overall subjects in He et al.’s study was relatively slower than ours due to differences in educational environments in different regions of China [45]. Furthermore, Cho et al. [9] reported that 46% of subjects (age range: 6 to 10 years) demonstrated slow myopic progression after being fitted with 6.0 mm-BOZD OK lenses (Menicon Z Night, Contactlenzen B.V., Emmen, Netherlands) for two years. Using different designs of OK lenses and the prolonged follow-up time may partly account for the discrepancy between results. These suggested that the proportion of slow progressors may be a useful comparative measure when evaluating the effectiveness of myopia controlling contact lenses.

Baseline myopia refraction is one of the influencing factors determining the myopia control effect, especially when using OK lenses [13, 34]. In an earlier study, children in the OK group with greater myopia at baseline had a smaller change in AL during the one-year follow-up [28]. For children wearing OK lenses, at each age, the probability of AL fast progression decreased as baseline myopia increased [46]. Based on these, we took baseline SER into account in the present study and found that only 5.0 mm-BOZD OK lenses and DISC lenses significantly delayed myopia progression in low myopes with baseline SER of − 1.00 D to − 2.50 D compared to the control group. Children fitted with 6.2 mm-BOZD OK lenses did not experience significant positive myopia controlling effects. On the other hand, with respect to children with moderate myopia (SER of − 2.50 D and over), 5.0 mm-BOZD OK lenses, 6.2 mm-BOZD OK lenses, and DISC lenses had comparable efficacy in slowing AL elongation. These results indicated that 8–11-year-old children with moderate myopia benefit more from traditional 6.2 mm-BOZD OK lenses than children with low myopia. This was supported by previous observations that OK lenses achieve notable peripheral myopic defocus in moderate to high myopes (SER: − 3.00 D to − 6.00 D) [4]. To achieve effective myopia control, 8–11-year-old low myopes with SER less than − 2.50 D would be better served by 5.0 mm-BOZD OK lenses or DISC lenses. However, as suggested by Queirós et al. [46], older myopic children with lower values of myopia still require close observation and vigorous intervention if their axial growth exceeds the physiological growth range (0.1 mm/year).

One accepted theory regarding how OK lenses retard myopia progression is the imposition of myopic defocus on the peripheral retina [47]. The degree of relative peripheral myopia after OK treatment usually increases with the extent of central myopia to be corrected, at least in patients with low and moderate myopia [48, 49]. The RCRP to the corneal apex can indicate the extent of myopic defocus induced by OK lenses on the peripheral retina [22, 50, 51]. Yang et al. have proposed that RCRP shifting closer to the central region may be more effective in retardation of myopia progression than that in the peripheral region after OK treatment [39]. Our previous study has found that maximum RCRP within the 4.8 mm-pupillary area did not significantly influence AL elongation after OK treatment [22]. Therefore, we compared the summed RCRP within the 4.8 mm-diameter (Sum4.8) in two groups and found that the Sum4.8 value in the 5.0 mm-BOZD OK group was significantly larger than that in the 6.2 mm-BOZD OK group. Furthermore, a larger pupil size may allow more peripheral defocus to fall within the pupil margin and therefore offers greater myopia control effect after OK treatment [52]. Based on this view, recent OK lens designs have aimed to decrease the treatment zone size and bring the mid-peripheral defocus ring closer to the pupil [21, 53]. Herein, we found that 5.0 mm-BOZD OK lenses produced a smaller treatment zone size and meanwhile formed a larger percentage of defocus zone within the pupil area in the cornea than 6.2 mm-BOZD OK lenses, indicating that a smaller treatment size induced by 5.0 mm-BOZD OK lenses may help the peripheral retina receive more myopic defocus signals. This could partly explain why the 5.0 mm-BOZD OK lenses were more effective in slowing AL elongation among 8–11-year-old children than 6.2 mm-BOZD OK lenses.

To produce different magnitudes of peripheral myopic defocus, SCLs were designed with different additions (+ 2.00 D to + 6.00 D) [15–17, 54]. One animal study proposed that + 5.00 D lenses, but not + 6.00 D and + 10.00 D lenses, had the highest effectiveness in inducing adequate myopic defocus in tree shrew eyes [55], suggesting that animals and humans can detect only a limited range of degrees of myopic defocus. Consistent with this finding, Huang et al. suggested that the addition of multifocal SCLs designed for inducing myopic defocus did not follow a “the higher, the better” principle, and that a defocus of + 6.00 D was ineffective for controlling myopia progression [54]. The effectiveness of DISC lenses in the present study and MiSight® lenses in other studies [17, 18, 25] indicates that bifocal SCLs with adds of + 2.00 D to + 2.50 D may achieve adequate myopic defocus. Moreover, the treatment zone size and the pupil diameter were also important factors influencing the myopia controlling effects of bifocal SCLs [23]. For DISC lenses, a 3.0 mm-central correction zone was even smaller than the treatment zone induced by 5.0 mm-BOZD OK lenses (average 4.3 mm in diameter), and therefore the near defocus rings were close to the pupil. It is expected that DISC lenses can lead to a sharp image point surrounded by a blur of rings on the retina. Above all, balancing between the correction zone and defocus zone, as well as producing enough defocus in the peripheral retina are essential considerations for the design of OK lenses and bifocal SCLs [21–23].

One limitation in our study is that duration of one year was relatively short. Long-term studies would answer whether the myopia controlling effects of 5.0 mm-BOZD OK lenses or DISC lenses in 8–11-year-old patients are sustained over time. The second limitation is that two different brands of OK lenses, Euclid and DRL, were used in the present study. Further validation is needed to determine whether the differences in the designs of the two lenses may partially contribute to the differences in the myopia controlling effect. Moreover, peripheral refraction, which reflects the extent of peripheral myopic defocus induced by the OK and DISC lenses, was not measured in this study. An appropriate method to describe and infer changes in the peripheral optical defocus should be applied in future studies. Furthermore, the present study failed to collect data on individual pupil size and the decentration for the DISC group. As the pupil size, lens decentration, and peripheral refraction of different individuals are probably different, the induced optical effects of the contact lenses may differ between individuals [23].

## Conclusions

In conclusion, 5.0 mm-BOZD OK lenses and DISC lenses effectively controlled myopia progression in 8–11-year-old children with myopia regardless of the diopter. However, 6.2 mm-BOZD OK lenses were more suitable for children with higher degrees of myopia (higher than − 2.50 D). These results indicate that each intervention has its own advantages for a given population. Based on our findings, clinicians may choose appropriate lenses for children with myopia to achieve ideal myopia control effects.

## Data Availability

The datasets generated/analyzed during the current study are available from the corresponding author on reasonable request.

## Abbreviations

AL: Axial length
ANOVA: Analysis of variance
BOZD: Back optic zone diameter
CRT: Corneal refractive therapy
Dk: Oxygen permeability
DISC: Defocus incorporated soft contact
DRL: Double reservoir lens
OK: Orthokeratology
RCRP: Relative corneal refractive power
SER: Spherical equivalent refraction
SCLs: Soft contact lenses
TD: Total lens diameter
VST: Vision shaping treatment
